# Trials in rare diseases: the need to think differently

**DOI:** 10.1186/1745-6215-12-S1-A107

**Published:** 2011-12-13

**Authors:** Lucinda Billingham, Kinga Malottki, Mark Pritchard, Neil Steven

**Affiliations:** 1 MRC Midland Hub for Trials Methodology Research, University of Birmingham, Birmingham B15 2TT, UK; 2 Cancer Research UK Clinical Trials Unit. University of Birmingham, Birmingham B15 2TT, UK

## Background

In general, large-scale clinical trials are needed to make definitive conclusions regarding changes in treatment practice. This is because treatment effects are often modest and large numbers of patients are needed to detect such differences with sufficient power for robust conclusions. Such trials are difficult and often impossible in rare diseases and their worth in this setting could be questioned and this drives the need to consider a different approach.

## Methods

A review is undertaken of methods that may be appropriate for making conclusions from clinical trials of treatment in rare diseases. In particular, the practicalities of applying a Bayesian strategy [1] are assessed by application to a trial in Merkel cell carcinoma.

## Results

It may be possible to run a traditional trial in rare diseases by adapting logistical aspects in order to maximise recruitment but alternative statistical approaches are normally needed. In rare diseases, if the focus is on estimation rather than hypothesis testing then important information can be gleaned from small trials, with interval estimates providing a measure of the uncertainty inherent in small studies. Using a Bayesian approach to trial design in the rare disease setting has been promoted as this enables information gathered from relevant previous studies to contribute to the estimation process. Proposed methodology suggests incorporating all levels of evidence, including case studies, into the prior distribution with appropriate weighting to reflect quality [1]. Practical application of this approach highlights that the substantial effort in incorporating lower levels of evidence may not be worth the gain in information, especially when such information is prone to bias.

## Conclusions

Treatment decisions in rare diseases should be based on evidence but the traditional approach to trials is difficult and therefore there is a need to think differently. Small trials focused on estimation rather than hypothesis testing are worthwhile as they will reduce the uncertainty about treatment effects. Incorporating prior knowledge together with trial data using a Bayesian approach can further reduce the uncertainty but the acceptability of this approach is subject to the believability of the prior information.
